# Feasibility and acceptability of insecticide-treated plastic sheeting (ITPS) for vector control in Papua New Guinea

**DOI:** 10.1186/1475-2875-11-342

**Published:** 2012-10-09

**Authors:** Justin Pulford, Anthony Tandrapah, Jo-An Atkinson, Brown Kaupa, Tanya Russell, Manuel W Hetzel

**Affiliations:** 1 Papua New Guinea Institute of Medical Research, PO Box 60, Goroka, EHP 441, Papua New Guinea; 2 The University of Queensland, School of Population Health, Herston, QLD 4006, Australia; 3 James Cook University, Faculty of Medicine, Health and Molecular Sciences, Cairns, Australia

## Abstract

**Background:**

This study assessed the feasibility and acceptability of utilizing insecticide-treated plastic sheeting (ITPS) as a malaria control intervention in Papua New Guinea (PNG).

**Methods:**

ZeroVector® ITPS was installed in 40 homes across four study sites representing a cross section of malaria transmission risk and housing style. Structured questionnaires were completed at the time of ITPS installation (n=40) and at four weeks post installation (n=40) with the household head. Similarly, group interviews with the male and/or female household heads were completed at installation (n=5) and four-week follow-up (n=4).

**Results:**

ZeroVector® ITPS was successfully installed in a range of homes employing traditional and/or modern building materials in PNG. The ITPS installations remained intact over the course of the four-week trial period and were highly acceptable to both male and female household heads. No dissatisfaction with the ITPS product was reported at four-week follow-up; however, the installation process was time consuming, participants reported a reduction in mosquito net use following ITPS installation and many participants expressed concern about the longevity of ITPS over the longer term.

**Conclusion:**

ZeroVector® ITPS installation is feasible and highly acceptable in a diverse range of PNG contexts and is likely to be favourably received as a vector control intervention if accessible en masse. A longer-term evaluation is required before firm policy or public health decisions can be made regarding the potential application of ITPS in the national malaria control programme. The positive study findings suggest a longer-term evaluation of this promising malaria control intervention warrants consideration.

## Background

Papua New Guinea (PNG) is a highly malarious country of approximately seven million people situated in the Western Pacific region. The country has a well-developed National Malaria Control Programme (NMCP) currently supported by a Round 8 grant from the Global Fund to Fight AIDS, Tuberculosis and Malaria (GFATM). Long-lasting, insecticide-treated mosquito nets (LLIN) are the mainstay of the PNG NMCP with plans to distribute over six million LLINs free of charge countrywide during the course of the Round 8 GFATM grant (2009–2014). However, a recently published evaluation of an earlier countrywide LLIN distribution campaign suggests achieving high (80% or more) LLIN coverage and usage rates across PNG will be challenging [1]. The NMCP further advocates the use of complementary indoor residual spraying (IRS) in the malaria epidemic-prone PNG Highlands region, yet the roll out of IRS in PNG remains irregular primarily due to operational constraints. Thus, additional methods of malaria control may be required to achieve a significant and sustained reduction in malaria in PNG [2].

Insecticide-treated plastic sheeting (ITPS), used as a wall covering, may provide a useful complementary vector control method in combination with, or as an alternative to other options such as LLINs and IRS. Similar to IRS, ITPS acts against indoor-resting mosquitoes; however, while IRS has to be applied once or twice a year, ITPS remains active for a three- to five-year period, similar to LLINs [3]. Vestergaard Frandsen (VF), a major supplier of LLINs, is the leading developer of ITPS. The VF product is a polyethylene lining with the insecticide deltamethrin incorporated into its fibres. Deltamethrin has been used widely in LLINs and has been found to be safe and effective [4]. Entomological studies have further demonstrated the effectiveness of deltamethrin-incorporated ITPS under a range of field conditions [3,5,6].

Previous studies with ITPS have shown good acceptability; however, they have focused on African and Asian settings [5,7]. Most PNG dwellings differ significantly from African houses (or the experimental huts designed for vector control efficacy trials) in terms of size, building materials and usage. They also show significant heterogeneity within PNG, e g, between highland and lowland areas, between traditional and modern buildings, or between very traditional and modernized social structures. Before considering potential applications of ITPS as a vector control intervention in PNG, preparatory studies are required to investigate the technical feasibility and acceptability of introducing ITPS across different settings. Accordingly, this paper presents findings from a largely qualitative inquiry designed to assess the operational feasibility of implementing ITPS in PNG and its acceptability in local communities. The primary research aims included: to identify possible constraints on affixing ITPS inside different types of Papua New Guinean dwellings; to identify factors that may impact ITPS durability once affixed; and, to explore community perceptions and acceptability of ITPS in different types of Papua New Guinean dwellings in various social and ecological settings.

## Methods

### Study sites and house selection

This study was conducted from February to April 2012 at the end of the wet season when malaria transmission is generally elevated. Four study sites were purposely selected on the basis that, collectively, they represented a cross section of malaria transmission risk and housing style. The selected study sites included a village in the non-endemic, but malaria epidemic-prone PNG Highlands, villages in the malaria endemic PNG lowlands and Islands regions and a suburb in the second largest town in the PNG Highlands region. Ten homes from each study site were selected for inclusion, in consultation with community leaders and following a community meeting in which the purpose of the study, risks and benefits, and eligibility criteria were outlined. The criteria for inclusion in the study were: that the housing structure was considered representative of housing at the study site as informed by community leaders; that the owner of the housing structure agreed to support ITPS installation within the following 48 hours; and that the owner of the housing structure agreed to maintain the ITPS in place for a period of no less than four weeks following installation.

### Procedure

#### ITPS installation

The ZeroVector® ITPS was installed in one bedroom in each home by trained research staff. Training was conducted on-site by an independent contractor, appointed and remunerated by Vestergaard Frandsen. Consistent with standard ZeroVector® ITPS installation protocols, each installation team consisted of three individuals and home owners were invited to assist if they wished. Installation materials included the ZeroVector® ITPS, nails of various sizes, nail caps, a craft knife, a 5 m tape measure, gloves and safety goggles. Installation followed a five-step process in which: 1) the home owner was asked to remove any wall hangings and potential obstacles from the bedroom in which the ITPS was to be hung, then the installation team measured and cut the required length of ITPS material; 2) the ITPS material was nailed to the internal wall, covering open eaves where possible; 3) the ITPS material was cut to expose door and window openings (if the homeowner chose not to use the ITPS as a window screen); 4) the ITPS was securely fixed around exposed door and/or window frames; and 5) home owners were advised of precautions and how to care for the ITPS material. The ITPS material installed in all homes was blue in colour.

#### Data collection

A structured checklist was completed at the time of ITPS installation by a member of the research team in consultation with the household head. Core characteristics of the house, installation time and resources were recorded on the checklist. The installation team leader also recorded a narrative of the installation process, inclusive of any problems experienced and/or anticipated. Then four weeks post installation, an interviewer-administered questionnaire was completed with the household head. Items on the follow-up questionnaire included participant perceptions of the installation process, ITPS aesthetics and durability, changes to the indoor environment, impact on mosquitoes and other insects and potential side effects. English and *Tok Pisin* versions of the follow-up questionnaire were available. A research team member completed a visual inspection of the ITPS installation at the four-week follow-up point. The visual inspection followed a structured format and focused on identifying signs of wear and tear in the ITPS product.

One male and/or female from each participating household was invited to participate in a group interview (GI) immediately following installation and at the four-week follow-up. The criteria for inclusion in the GIs were: that he/she must normally reside in a housing structure in which the ITPS was installed; that he/she must be the male/female household head or have been authorized by the household head to speak on his/her behalf; and that the household representative must be 18 years of age or older. All GIs followed a schedule variously focusing on initial and subsequent impressions of ITPS, installation and ‘user’ experiences, and the expected and realized advantages and disadvantages of ITPS, including real or perceived side effects.

#### Data analysis

All quantitative data were entered onto an Excel spreadsheet. Descriptive statistics were conducted as required. All interviews were recorded on a digital voice recorder, transcribed verbatim, translated into English, and entered into NVIVO 9. A thematic analysis of the interview data was conducted as informed by a general inductive methodology [8]. Interview data were independently coded by two investigators (JP, JA). Initial codes were derived from the research aims and were subsequently refined over two coding cycles. The two coders compared and agreed upon codes and emerging themes at the end of each cycle, resolving disagreement by consensus opinion or by the creation of new, mutually agreeable, codes/themes.

#### Ethical aspects

This study was granted ethical clearance by the Medical Research Advisory Committee of PNG (MRAC No. 11.22; December 2011). Following verbal and written explanation of study aims and procedures, informed consent (written or witnessed thumb print) was obtained from the household head of all homes in which ITPS was installed and from all GI participants. As an incentive for participation, household heads were given the option of retaining the ITPS product following the completion of the study.

## Results

### Sample

**Figure 1:**
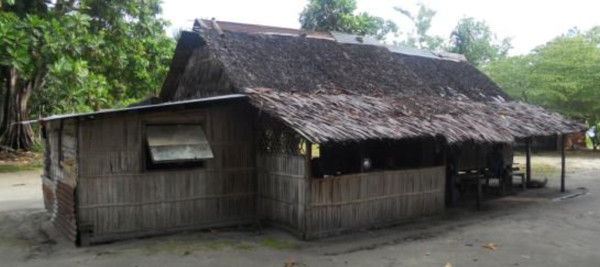
A traditional housing structure in Lokwitua, PNG Islands.

**Figure 2:**
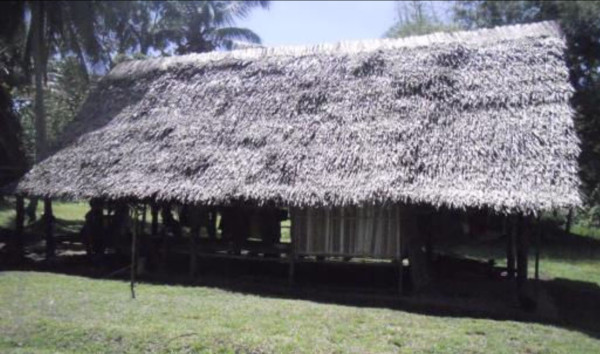
A traditional housing structure in Nauna, PNG lowlands.

**Figure 3:**
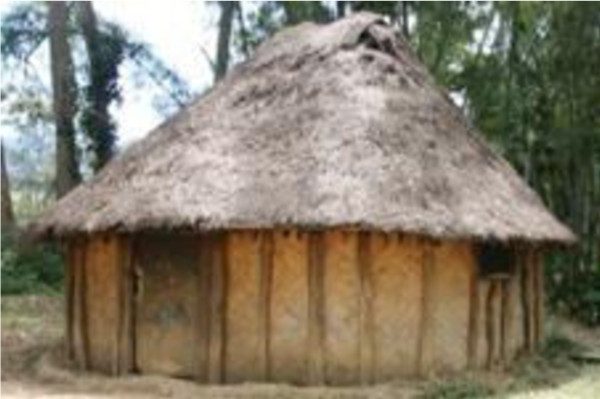
A traditional housing structure in Masumave, PNG Highlands.

**Figure 4:**
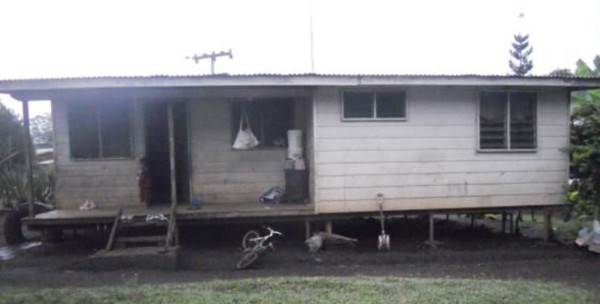
A home constructed out of commercial materials in Coffee Roots, PNG urban.

Ten homes from each of the four study sites were included in the trial. Selected characteristics of these homes, by study site and overall, are presented in Table 1. As shown, 52.5% of the homes were entirely constructed of traditional materials. Bamboo and/or untreated/unprocessed timber was used for the structural framework, bamboo, *pitpit* (coarse tubular grass similar to bamboo) and/or sago palm formed the internal and external walls and thatched grass or sago palm leaves were used for the roofing material. Figures 1, 2, 3 present typical examples of traditional housing from three of the study sites. A quarter of the homes (25%) were constructed entirely of commercially available materials such as plywood, iron sheeting and treated/processed timber (see Figure 4) and a further 22.5% were constructed of both traditional and commercial materials.

**Table 1:** Median number of residents per household, rooms per household, rooms used for sleeping per household and type of housing material by study site

**Study Site**	**Residents per household**	**Rooms per household**	**Bedrooms per household**^**1**^	**Type of housing material Trad**^**2**^**. Mixed**^**3.**^**Comm**^**4**^**.**		
	**Median (range)**	**Median (range)**	**Median (range)**			
Islands village	3.5 (2, 7)	2 (1, 3)	2 (1, 3)	70%	30%	0%
Lowlands village	5 (3, 10)	3 (2, 5)	3 (1, 5)	80%	10%	10%
Highlands village	3 (1, 6)	1 (1, 4)	1 (1, 4)	60%	30%	10%
Highlands urban	6.5 (3, 11)	4.5 (2, 6)	4 (2, 5)	0%	20%	80%
**Overall**	**5 (1, 11)**	**3 (1, 6)**	**3 (1, 5)**	**52.5%**	**22.5%**	**25%**

1 Includes rooms exclusively used for sleeping and rooms used for sleeping and other activities. 2 Trad. = traditional. 3 House comprised of a mix of traditional and commercial materials. 4 Comm. = commercial.

The installation checklist was completed for all participating households (n=40). The follow-up questionnaire was completed with one household head, either male or female, from all 40 homes. Nine GIs were completed with a combined total of 63 participants. Informational redundancy was achieved within the nine GIs and hence no further recruitment of participants was found to be necessary. Five of the GIs were completed immediately post-installation (two gender-separated GIs were conducted in the lowlands village, one mixed-gender GI was conducted in each of the three remaining sites) and four at follow-up (one in each site). Table 2 presents the number and sex of GI participants by study site.

**Table 2:** Number and sex of group interview participants by study site

**Study Site**	**Installation**			**Follow-Up**		
	**Total**	**Male**	**Female**	**Total**	**Male**	**Female**
Islands village	6	2	4	8	1	7
Lowlands village	12	6	6	9	4	5
Highlands village	7	2	5	6	2	4
Highlands urban	9	4	5	6	4	2
Overall	34	14	20	29	11	18

### ITPS feasibility

**Figure 5:**
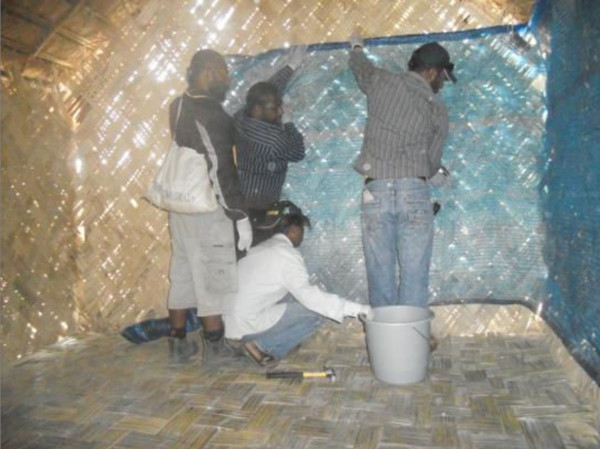
Installing ZeroVector® ITPS in a Highlands village house.

The installation teams were able to install the ZeroVector® ITPS in one bedroom in all 40 homes. Table 3 presents the median length of ZeroVector® ITPS material used per bedroom, the median number of nails required and the median installation time in each study site and overall. Figure 5 depicts a ZeroVector® ITPS installation in progress.

**Table 3:** Median length of ZeroVector® ITPS material utilised per room, number of nails utilised per room and installation time per room by study site

**Study Site**	**Material length (cm)**^**1**^	**No. Nails**	**Installation time (min)**
	**Median (range)**	**Median (range)**	**Median (range)**
Islands village	1200 (900, 4800)	92 (63, 168)	83.5 (43, 123)
Lowlands village	1100 (900, 1300)	68 (44, 79)	64 (27, 85)
Highlands village	1400 (900, 1600)	102 (50, 163)	121 (69, 203)
Highlands urban	1150 (850, 1400)	88.5 (72, 110)	79 (50, 118)
Overall	1200 (850, 4800)	81.5 (50, 168)	82.5 (27, 203)

1. Standard width of ZeroVector® ITPS = 240cm.

A number of challenges were identified by the research team during the installation phase. The diversity in house size, shape and construction within village settings, made it difficult to establish a ‘routine’ installation process. The external support beams in homes constructed out of traditional building materials were typically rough cut logs which rarely ran flush with the external/internal wall lining (note external beams in Figure 3). As such, affixing a nail into these beams was often difficult and, because the external/internal wall lining was typically made of *pitpit* or bamboo with a width of usually no more than 5 mm, a secure installation required the ITPS to be nailed firmly into the external support beam. Locating the external support beam from inside the dwelling was also problematic and required a fourth person (in addition to the three members installing the ITPS) to stand outside the home to direct the placement of nails. Finally, in many of the traditional homes the bed stands and tables were embedded in the (pressed dirt) floor (thus, not easily removable) and pressed against the interior wall, rendering it difficult to hang the ITPS in these areas.

**Figure 6:**
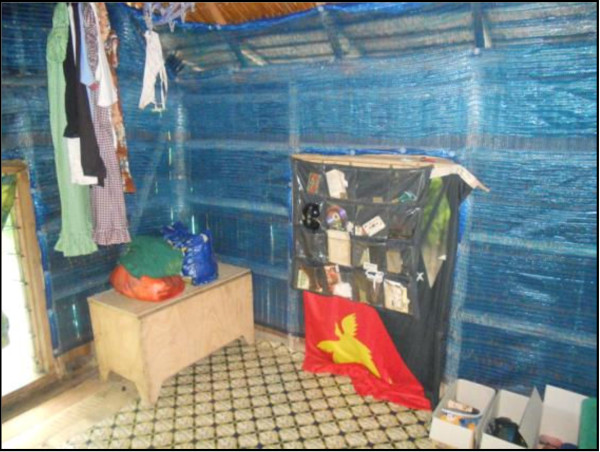
The interior wall of an Islands village house four-weeks post ZeroVector® ITPS installation.

The ITPS material remained as installed in all 40 homes at the four-week follow-up (i e, no material had been removed or had become unattached during this time). All nails affixed by the installation team were still firmly in place in 37/40 homes. In three homes a maximum of two nails had worked loose. In no cases had home owners added further fixtures to strengthen the ITPS installation. The ITPS material remained completely intact in 38/40 homes. In one home, two holes in the ITPS material had reportedly been made by a rat and in another a hole had been made in the ITPS material attached to the door frame as a result of friction when the door was opened or closed. One or more objects such as a bed, table or picture was observed to be in direct contact with the ITPS material in 19/40 homes (as depicted in Figure 6). There was no evidence of any deterioration in the ITPS material at any of these contact points (e g, the point where a bed frame was in direct contact with ITPS material) in any of these 19 homes.

### ITPS acceptability – follow-up survey findings

Five (5/40) participants reported experiencing some concerns about having the ITPS installation team enter their home. Reported concerns included embarrassment about current living situation (n=3), anxiety about the possible side effects of ITPS (n=2), and apprehension at not being present when the installation team was in the home (n=1).

All participants (40/40) reported a perceived reduction in the number of mosquitoes in and around the house and a reduction in the number of mosquito bites following ITPS installation. Thirty-nine participants (39/40) reported a reduction in other insect species and 11/40 participants reported a reduction in rodents.

Three participants reported that one or more household members experienced ill health after the installation. One of these participants explicitly stated the illness was not related to the installation, another reported that a number of family members experienced flu-like symptoms, and one participant reported another family member having experienced a malaria episode.

Twenty-five participants reported a perceived change in the inside temperature following ITPS installation. In 19/25 cases the perceived change was an increase in temperature and in 6/25 cases a decrease in temperature (n-b, 12 of the 19 participants that reported an increase in temperature were from the cooler highlands region, where the temperature increase was considered a positive side effect). Twenty-one participants reported that the room was darker following ITPS installation and a further 15 reported an unusual smell following ITPS installation. The smell was typically described as that of insecticide and in all cases was reported as no longer perceptible at the time of follow-up interview.

Thirty participants reported that they liked the blue colour of the ITPS material; 10 did not. A range of colour preferences was identified, although the most frequently expressed preferences were for blue (n=20) or lighter ‘bright’ colours (n=8). The most frequently expressed least preferred colours were ‘dark’ colours as they would darken the room (n=17) or white or ‘light’ colours (n=9) as they would more readily reveal dirt or dust. No participant suggested a colour that would be either favourable or unfavourable for customary or cultural reasons.

Thirty-four participants reported that the ITPS material enhanced the aesthetics of their home in some way. Thirty-seven participants reported that one or more visitors had witnessed the ITPS installation; all reported that the visitors considered it favourably. All participants opted to retain the ITPS installation at the four-week follow-up.

### ITPS acceptability – group interview findings

Analysis of the GI transcripts indicated high acceptability of the ITPS product amongst all participants, across all study sites. The high acceptability was seemingly influenced by five key factors. Firstly, many GI participants reported observing insects dying during, or immediately following, ITPS installation.

"‘The first day after [ITPS] installation I saw mosquitoes flying into the house, contact the material and then just fall off and die. The cockroaches climbed up the [ITPS covered] wall and died instantly.’ (Lowlands village, installation GI, male)."

Witnessing the ITPS working so quickly and effectively led to the early formation of a positive impression, as did the apparent repellent effect of ITPS on larger pests such as rats and mice. Secondly, the observed impact of ITPS on mosquitoes and other insects translated into better nights’ sleep as a result of the reduction in mosquito nuisance, further enhancing the positive impression formed:

"‘I do not see any more cockroaches, rats or ants inside my house…we are now sleeping peacefully inside the room.’"

"(Islands village, follow-up GI, female)."

Thirdly, the initial effectiveness of the ITPS was sustained over the four week period,whilst the anticipated ‘risks’ (discussed in detail below) failed to materialize.

"‘The [ITPS] is still effective because the insects just disappear…the insects just inhale the smell and became unconscious.’"

"(Highlands urban, follow-up GI, male),"

The fourth key influence on ITPS acceptability pertained to the product aesthetics, rather than its function as an insecticidal intervention. Most participants felt that the blue ITPS material enhanced the internal appearance of their home as exemplified in the following excerpt:

"‘When I opened the door and went into the house it looked a lot different [following ITPS installation]. My house looked beautiful and was glowing.’"

"(Highlands urban, installation GI, male)."

Many participants also noted that the ITPS acted as an additional building material by blocking holes in the existing walls, reducing draughts, noise levels and the amount of dust entering the home. Finally, the high acceptability of the ITPS product among study participants was seemingly influenced by a level of prestige associated with ‘owning’ a new and largely unavailable (to non-study participants) malaria control intervention. This prestige value was perhaps most evident when GI participants discussed the reactions of non-household members to the ITPS product, which were invariably positive and suggestive of a degree of envy. For example,

"‘One of my sisters came and saw the durable lining sheets and liked it and said she wished she could have got one like this too.’"

"(Islands village, installation GI, female)."

Whilst all five of the aforementioned factors contributed to the high acceptability of ITPS among GI participants, the primary influence on acceptability was the observed effectiveness of the insecticidal properties of the ITPS and the protection this confers against malaria and other vector-borne disease. This is perhaps best exemplified in the following quote:

"‘We do not want to be sick with malaria. If the kids are sick, we will struggle to walk a long way to go to the hospital. We do not want this to happen. This plastic sheeting will help protect us and our children from getting sick with malaria.’"

"(Lowlands village, follow-up GI, male)."

No GI participant expressed dissatisfaction with the ITPS installation at the four-week follow-up. However, a number of issues emerged that warrant consideration for future ITPS trials in PNG or elsewhere. Firstly, a number of participants expressed concerns about the ITPS product at the time of installation and a smaller number expressed concerns about the installation process itself. The primary concern was the possibility of side effects from the insecticide used in the ITPS product, especially among small children:

"‘I have a small child and I was worried that the insecticide on the durable lining sheet might have a bad effect on my child.’"

"(Islands village, installation GI, female)."

Other expressed concerns included the potential flammability of the product, the appearance of the product and the possibility of theft (by the installation team) during the installation process. Whilst the perceived risks had not materialised at the four-week follow-up and the concerns regarding installation did not prevent installation, the presence of such concerns pose a potential threat to ITPS acceptability and/or utilisation if not properly managed.

As previously noted, the ITPS product sustained no damage in most study homes during the four-week trial period and the installation remained securely fastened in all homes. Nevertheless, many participants perceived the product to be relatively fragile and expressed some concern about its potential longevity over an extended period of time:

"‘The material is not strong. It is just made of plastic.’"

"(Highlands urban, follow-up GI, female)."

A number of participants even reported taking specific precautions to avoid damaging the product,

"‘When the children go inside the house I always shout to them [reminding them not to touch the ITPS material], they might forget and pull it and then it will get loose.’"

"(Lowlands village, installation GI, female)."

These precautions are unlikely to be maintained over time, especially as the material starts to discolour, degrade or lose its perceived effectiveness.

Mosquito net use prior to ITPS installation was variable in all study sites, with many participants reporting little or inconsistent use. The ITPS product reportedly reduced mosquito net use even further as exemplified by the following quote:

"‘I only used the mosquito net when I see a lot of mosquitoes around. After the installation of the plastic sheeting I do not bother to use the net anymore.’ (Islands village, follow-up GI, female)."

Similar instances of reducing or ceasing mosquito net use as a result of a perceived reduction in mosquito numbers following ITPS installation were reported across all study sites. Many participants considered the ITPS to be a superior malaria control intervention compared to mosquito nets and this perception further contributed to the subsequent reduction in net use:

"‘They have distributed [long-lasting insecticide treated] mosquito nets and informed us that it will kill the mosquitoes, but we could still see the mosquitoes flying and buzzing around. When using the plastic sheeting we could practically see the mosquitoes and other small insects falling to their death.’"

"(Highlands village, follow-up GI, female)."

## Discussion

The findings presented in this paper indicate that ZeroVector® ITPS can be successfully affixed in a range of homes employing traditional and/or modern building materials in PNG. The findings further indicate that ZeroVector® ITPS is durable over the short-term and is highly acceptable to male and female householders residing in malaria endemic and epidemic prone regions of PNG. Thus, on the basis of this trial, ZeroVector® ITPS presents as a potentially viable vector control intervention in a PNG context. However, this study also raised a number of issues pertaining to the installation and utilization of ITPS that warrant careful consideration from a policy, public health and research perspective.

The first of these issues pertains to the process of installing ITPS in traditional homes, which presents as a particularly time-consuming process. If ITPS were to be installed in every sleeping room within a typical dwelling, as has been suggested to achieve maximum health impact [3], then one might expect the median installation time per home in PNG to be just over four hours, excluding preparation time (given the median number of sleeping rooms per dwelling across all four study sites was three and the median installation time per room was 82.5 minutes). An installation time of this magnitude suggests the widespread application of ITPS may require householders or communities to take responsibility for local ITPS installation as the cost of contracting specialist teams to complete the task would likely be prohibitive. An installation model of this nature would contrast with traditional IRS and LLIN distribution campaigns where the demands placed on household members and communities are relatively minimal. Thus, if widespread implementation of ITPS were to be considered as an alternative or complement to LLIN or IRS, then careful consideration would have to be given to programme roll out. Key questions might include: what level of training and support would community members require? How could the relatively bulky ITPS rolls (standard roll is 100 m in length and 2.4 m in width) and supporting resources (e g, nails, hammers, tape measures) be reliably and cost effectively transported to often remote locations? How would the uptake and quality of product installation be monitored?

The second issue of note pertains to the potential impact of ITPS on LLIN utilization. The installation team were careful to advise all participants that they should continue to use their LLINs following ITPS installation; however, many participants reportedly ignored this advice and ceased or reduced mosquito net use. The observed effectiveness of the ITPS and the resulting reduction in insect numbers contributed to this change in behaviour and it is likely that other factors contributed to this decision as well. The ITPS, once installed, requires little effort to maintain on the owner’s part and does not encroach on living space. Lack of space and attitudinal indifference have previously been reported as barriers to mosquito net use in PNG [9]. Thus, the convenience and space maximizing properties of ITPS relative to LLINs may further reduce (the often already low) motivation to reliably use a mosquito net.

Evidence indicates that ITPS offers considerably less personal protection against malaria vectors when compared to LLINs [10], so an individual who foregoes net use following ITPS installation may place his or her self at greater risk of malaria infection. Thus, introducing ITPS in an LLIN-owning household could potentially increase the risk of malaria infection at an individual level. Furthermore, combining two long-lasting, deltamethrin-based, vector-control interventions, such as ITPS and LLIN, could potentially accelerate the development of insecticide resistance [11]. However, if an individual and/or his/her family do not use an (available) LLIN on a regular basis anyway, as has been documented in many malarious countries [12,13], then an ITPS installation may provide an alternative long-lasting vector control intervention; particularly if ITPS was installed in enough homes to afford community level protection. A conservative approach may be to employ ITPS as an alternative to IRS in areas where LLIN are not widely used. This would limit the potential for accelerated development of insecticidal resistance and would have minimal impact on LLIN use (as LLIN would not be widely available). The malaria epidemic-prone PNG Highlands region potentially presents as one such location in which this approach could be tested. Nevertheless, exploring potential uses of ITPS in other settings, ideally in conjunction with other complementary malaria control tools, should not be discounted as history has shown that over-reliance on a small number of malaria control tools can be problematic [14]. For example, experimental evidence indicates that the combined use of ITPS and LLIN, when each employs a different insecticide, affords a greater level of personal protection than either product used in isolation [15,16]. Given the potential value of ITPS as an additional malaria control tool, further research is needed to identify how it may be best applied in different epidemiological and cultural contexts. Particularly important to policy makers would be data on the effectiveness of ITPS in specified settings, the cost-effectiveness of employing ITPS in conjunction with or as an alternative to other malaria control interventions and the potential impact of ITPS on the development of insecticide resistance.

Whilst there were no significant issues with ITPS durability and/or the robustness of the installation during the four-week trial, many participants expressed concern about the longevity of ITPS over the longer term. A number of household items were observed to be in direct contact with the ITPS product which could potentially lead to friction-related wear and tear over time and the potential for children to mishandle the ITPS material was noted by participants, a problem previously identified as a source of damage to LLIN [9,17]. The ITPS installation was also exposed to smoke from internal, unventilated, fires in many of the traditional homes, which may degrade the material over time, reducing the aesthetic appeal and potentially impacting on insecticide longevity and potency. Traditional homes in PNG are also not permanent constructions, wall and roofing materials are regularly replaced and entire houses rebuilt after a period of time (typically between five to 15 years). Depending on the age and state of a home at the time of installation, the home owner may substantially repair or replace the home prior to the expected three to five-year duration of ITPS. It would be of interest to monitor how ITPS is managed in such a repair/rebuild situation, especially if some degradation in ITPS appearance and/or real (or perceived) effectiveness have occurred. All of these factors could potentially impact the mid-to-longer term feasibility, acceptability and utilization of ITPS in PNG. Accordingly, a sound evaluation of ITPS installation, especially a detailed cost-effectiveness analysis relative to alternative interventions such as IRS or LLIN, requires long-term monitoring of the ITPS installation over its expected lifetime.

The need for a longer-term evaluation of ITPS highlights the primary limitation of this study; namely, the relatively brief four-week duration. A study of this length was adequate to assess the feasibility of ITPS installation in diverse PNG settings and initial acceptability of the ITPS product. However, to thoroughly investigate household response to ITPS and to reliably determine the cost-effectiveness of employing ITPS on a larger scale, longer-term monitoring and evaluation of ITPS in a range of community settings is required. A further limitation of the current study is the possibility that participant responses to both the follow-up questionnaire and GI were influenced by social desirability bias. Participants were generally grateful to have been included in the trial and, as such, may have been reluctant to express criticism towards the ITPS which was effectively ‘gifted’ to them by the research team. Having said this, participants were strongly encouraged to express any criticisms they might have of the product and were given many opportunities to do so. Furthermore, all participants chose to retain the ITPS product suggesting that, even if left unexpressed, any dissatisfaction was likely to be relatively minor and insufficient to deter continued ITPS use.

## Conclusion

The study findings indicate that ITPS installation is feasible and highly acceptable in a diverse range of PNG contexts and is likely to be favourably received as a vector control intervention if accessible en masse. However, a longer-term evaluation of ITPS installations in community settings is required before firm policy or public health decisions can be made. Areas that require further investigation include: the optimal model of ITPS installation in the context of a large scale implementation programme, the impact of ITPS on LLIN use, the cost-effectiveness of ITPS *vs* LLIN or IRS campaigns based on long-term evaluation of ITPS over its effective lifetime, and the potential impact of ITPS on the development of insecticide resistance. The positive study findings suggest further evaluation of the ITPS product warrants consideration in the PNG context.

## Competing interests

The ZeroVector® ITPS product and training in its application were provided to this study free of charge by Vestergaard Frandsen. The authors declare that they have no competing interests.

## Authors’ contributions

JP contributed to study design, conducted the analysis and drafted the final manuscript, AT facilitated site selection and critically revised final manuscript, BK coordinated and conducted data collection, JA contributed to study design, data analysis and critically revised the final manuscript, and TR and MWH contributed to study design and critically revised the manuscript. All authors read and approved the final manuscript.
